# “Under pressure”: should we use diaphragm excursion to predict weaning success in patients receiving pressure support ventilation?

**DOI:** 10.1186/s13054-023-04504-8

**Published:** 2023-06-15

**Authors:** Emma Sabourin, Christophe Carpentier, Christopher Lai, Xavier Monnet, Tài Pham

**Affiliations:** 1 Service de Médecine Intensive-Réanimation, Hôpital de Bicêtre, DMU CORREVE, FHU SEPSIS, Groupe de Recherche CARMAS, Hôpitaux Universitaires Paris-Saclay, AP-HP, 94270 Le Kremlin-Bicêtre, France; 2 INSERM UMR S_999, Pulmonary Hypertension: Pathophysiology and Novel Therapies, University Paris-Saclay, Hôpital Marie Lannelongue, Le Plessis-Robinson, France; 3 INSERM U1018, Equipe d’Epidémiologie Respiratoire Intégrative, CESP, Université Paris-Saclay (UVSQ)—Université Paris-Sud, 94807 Villejuif, France

We read with great interest the recent article published in Critical Care by Huan Ma et al. entitled “Using automatic speckle tracking imaging to measure diaphragm excursion and predict the outcome of mechanical ventilation weaning” [1]. Diaphragm ultrasound is an interesting technique to better understand weaning physiology and outcomes, and although we agree with the authors' perspective, we think that the results of the study should be interpreted with caution.

This prospective, multicenter, observational study aimed to evaluate the ability of diaphragm excursion (assessed with automatic speckle tracking) to predict weaning outcome. The authors found a significant correlation between the automatic measurement of mean excursion and velocity in speckle tracking imaging and its manual measurement. After analyzing the receiver operating characteristic (ROC) curve, they showed that diaphragmatic velocity and mean excursion were promising high diagnostic values for prolonged weaning. Yet, the diagnostic value of diaphragm excursion was moderate for predicting in-hospital death, withdrawal of treatment and weaning failure.

We would like to raise a few points that appear important when using ultrasound to evaluate diaphragm function and that might impact the interpretation of the study.

First of all, diaphragm excursion was measured in patients receiving invasive mechanical ventilation in pressure support mode with support set at 10–12 cm H_2_O. It appears to us that the value of assessing diaphragm excursion under assisted mechanical ventilation, such as in this study, should be subject to caution and cannot be interpreted as the patient’s own respiratory muscle strength.

Indeed, as well demonstrated in several studies [2–4], it is not possible to differentiate which part of the diaphragmatic excursion measured is due to the external force applied by the ventilator (passive), and which part of the excursion is due to the diaphragmatic contraction (active). Measures of diaphragm excursion under assisted mechanical ventilation will consequently be overestimated because the patient’s diaphragmatic contraction is added to the passive excursion generated by the ventilator in pressure support mode.

Conceptually, this would not allow for weaning outcome prediction, and in their study, Zombon et al. [2] emphasized the fact that diaphragm excursion measures should be limited to patients with spontaneous breathing, as it is not a marker per se of diaphragm contraction or respiratory effort but a marker of diaphragm movement is highly dependent on inspired volumes.

M. Llamas-Álvarez and co-workers [3] also highlighted the fact that diaphragm excursion is only relevant in patients without ventilator support, and showed that diaphragm excursion interpretation entails several biases as diaphragm excursion may vary depending on several parameters such as the patient’s positioning, and thoracic or abdominal pressure variation. These authors even concluded that diaphragm excursion should not be used to assess diaphragm function.

Using diaphragm excursion to predict weaning success should therefore be measured in patients undergoing a spontaneous breathing trial, such as a T-piece trial (disconnecting the patient from the ventilator) or a ZEEP trial (decreasing the pressure support to minimal values with PEEP set at 0 cm H_2_0). In this situation, the excursion measured will hence apprehend the diaphragmatic contraction without the impact of the pressure support generated by the ventilator.

Diaphragm thickening fraction, which is also measured with ultrasound, is another interesting technique as several studies demonstrated its reliability to predict extubation success [4]. It might even be superior to diaphragm excursion in this indication: two studies have demonstrated a significant correlation between the diaphragmatic tidal thickening fraction and the diaphragmatic pressure–time product in patients receiving noninvasive ventilation after extubation and in healthy subjects and intubated patients with pressure support ventilation [4].

Moreover, some studies have shown an interesting and feasible method for predicting weaning success using the measurement of the right diaphragm thickening fraction in combination with the rapid shallow breathing index (RSBI). This combination has been shown to improve the precision of successful weaning prediction when compared with RSBI alone [5].

Therefore, the prediction of weaning success in patients undergoing assisted breathing trials should be evaluated by diaphragmatic thickening fraction as it is less impacted by pressure support variation [2].

In a nutshell, we think diaphragm excursion measurement is an interesting approach but should be done in patients with spontaneous breathing without pressure support to be able to predict weaning success. Nonetheless, this study has shown promising results regarding the feasibility and reliability of speckle tracking imaging, with high correlation values. Further research on diaphragm function assessment to predict weaning outcome is needed.

## Data Availability

Not applicable.
